# Genetic analysis of non-syndromic peg lateralis using whole-exome sequencing

**DOI:** 10.3389/fgene.2025.1572966

**Published:** 2025-08-13

**Authors:** Junglim Choi, Sungnam Kim, Hyunsoo Ahn, Donghyo Kim, Sung-Won Cho, Sanguk Kim, Jae Hoon Lee

**Affiliations:** ^1^ Department of Advanced General Dentistry, School of Dentistry, Dankook University, Cheonan-si, Chungcheongnam-do, Republic of Korea; ^2^ Department of Prosthodontics, Yonsei University College of Dentistry, Seoul, Republic of Korea; ^3^ Department of Life Sciences, Pohang University of Science and Technology, Pohang-si, Gyeongsangbuk-do, Republic of Korea; ^4^ Graduate School of Artificial Intelligence, Pohang University of Science and Technology, Pohang-si, Gyeongsangbuk-do, Republic of Korea; ^5^ Department of Chemical Engineering, Pohang University of Science and Technology, Pohang-si, Gyeongsangbuk-do, Republic of Korea; ^6^ Division of Anatomy and Developmental Biology, Department of Oral Biology, BK21 FOUR Project, Yonsei University College of Dentistry, Seoul, Republic of Korea; ^7^ Institute of Convergence Research and Education in Advanced Technology, Yonsei University, Seoul, Republic of Korea

**Keywords:** non-syndromic peg lateralis, whole-exome sequencing, genetic analysis, dental anomalies, calcium flux, RP11-131H24.4, OTOP1

## Abstract

**Introduction:**

Although peg-shaped lateral incisors are a common dental anomaly, the genetic mechanisms governing peg lateralis are poorly understood, particularly in cases where other associated anomalies are absent. Here, we aimed to identify potential candidate genes contributing to the development of non-syndromic peg lateralis via whole-exome sequencing (WES).

**Methods:**

Saliva samples were collected from 20 unrelated Korean individuals with non-syndromic peg lateralis. WES was conducted on these samples, and variants with *p*-value <0.05, false discovery rate <10^–10^, and odds ratio >1 were filtered. In-silico mutation impact analysis was performed using Polymorphism Phenotyping v2, sorting intolerant from the tolerant, and integrated score of co-evolution and conservation algorithms.

**Results:**

We identified a heterozygous allele for *RP11-131H24.4* and *OTOP1*, which encodes the otopetrin-1 protein, a proton channel, in all 20 individuals. Gene ontology analysis revealed an association between candidate genes and peg lateralis. We further confirmed that the peg lateralis candidate variants of the same genotype were found in the family members of three individuals.

**Conclusion:**

The results suggest a possible function of these newly identified genes in the development of peg lateralis, which remains to be defined. This study may provide new insights into the genetic basis of non-syndromic peg lateralis, establishing a basis for the further analysis of the disease-associated genes identified herein.

## 1 Introduction

More than 350 genes, most of which encode signaling factors with crucial roles in molecular and cellular interactions, have been found to be involved in odontogenesis (Townsend et al., 2012). Epithelial–mesenchymal interactions mediated by these proteins regulate the different stages of tooth formation, including initiation, morphogenesis, and differentiation (Tucker and Sharpe, 2004). Tooth anomalies, which appear as alterations in the number, size, morphology, or structure of a single tooth or multiple teeth, occur in isolation or in association with other syndromes (Cobourne and Sharpe, 2013). Congenital tooth anomalies cause functional, aesthetic, and psychological problems, sometimes leading to substantial facial changes (Suda et al., 2010; Taju et al., 2018; Townsend et al., 2005). Thus, early diagnosis is essential to allow for timely intervention, highlighting the need for studies on biomarkers of dental abnormalities.

**FIGURE 1:**
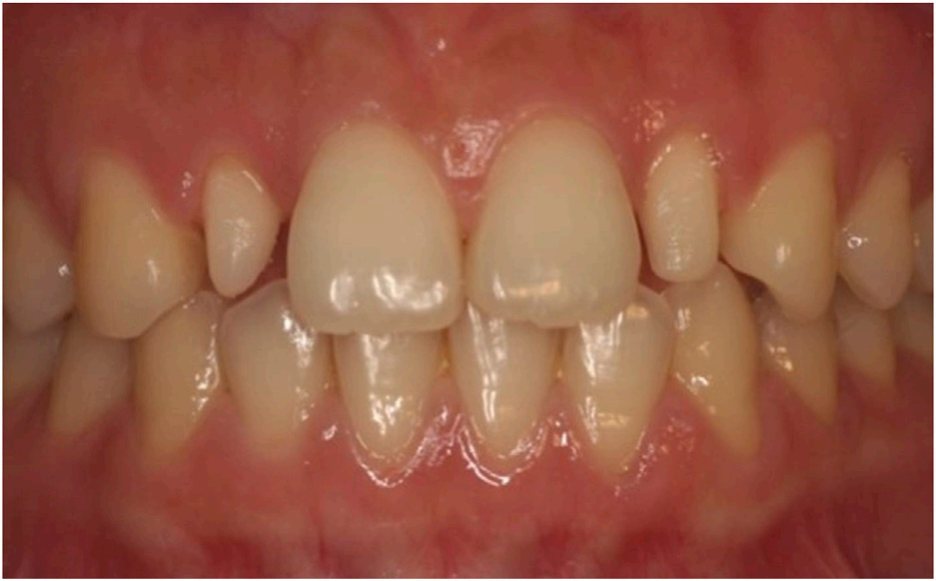
Peg lateralis on both maxillary lateral incisors.

In 1987, Granhene defined peg lateralis as a tooth with an incisal mesiodistal width shorter than the cervical width (Figure 1). The maxillary lateral incisors are often affected, presenting as tapered-shaped teeth, thus giving rise to the name peg lateralis (Davis, 1987). The prevalence of peg lateralis varies from 0.6% to 9.9% depending on ethnicity, sex, and region; Mongolians exhibit a significantly higher prevalence rate than other ethnic groups. According to meta-analyses, the overall prevalence rate is 1.8% (Hua et al., 2013). Although non-syndromic teeth abnormalities are inherited as an autosomal trait, existing literature suggests that peg-shaped teeth are 1.35 times more prevalent in women than in men (Polder et al., 2004). Both unilateral and bilateral peg lateralis seem to have a similar prevalence; however, the frequency of left-side unilateral peg lateralis is almost twice that of right-side ones (Magnusson, 1977; Meskin and Gorlin, 1963).

Furthermore, peg lateralis is associated with other tooth anomalies, such as hypodontia, palatal displacement of maxillary canines, dens invaginatus, tooth transposition, premolar rotations, and supernumerary teeth. These anomalies are considered to have the same genotype, presenting different phenotypes (Baccetti, 1998). In his etiological model, Brook suggested a relationship between alterations in number and size (Brook, 1984). These associations have been considered as different phenotypes of an identical genotype, leading to variations in tooth number and size. Brook suggested that the absence of a tooth may be a quasi-continuous phenotype of tooth size distribution (Brook, 1984; Liuk et al., 2013). Dental diseases are not determined by a single gene but by the interaction of various genes and environmental factors. Previous studies have identified mutation in genes, such as *MSX1, AXIN2, FGF3, EDA, WNT5A, WNT10A*, and *Pax9,* as related to the occurrence of both microdontia and hypodontia (Brook et al., 2014; Cai et al., 2011; Laurikkala et al., 2001). This pleiotropic and multifactorial nature of tooth anomalies, attributed to the complex mechanism of odontogenesis, makes it difficult to identify a single gene involved in a tooth anomaly. Owing to these obstacles, genetic studies of non-syndromic isolated peg lateralis have rarely been reported.

Herein, we analyzed peg lateralis without any syndromes or other tooth anomalies to gain further insight into the associated genotypes. To this end, we analyzed DNA from individuals with a peg-shaped incisor via whole-exome sequencing (WES), focusing on the protein-coding part of the genome, which contains approximately 85% of disease-related genes (Rabbani et al., 2014).

## 2 Materials and methods

### 2.1 Sample collection and DNA extraction

The study protocol was approved by the Institutional Review Board of Yonsei University College of Dentistry (Yonsei IRB No. 2-2020-0045). The individuals were recruited from August 2020 to August 2022 at Yonsei University Dental Hospital and Dankook University Dental Hospital. The experimental group consisted of 20 individuals with peg lateralis, regardless of gender and age. All individuals provided written informed consent to participate in this clinical trial, and parental consent was obtained for participants under 20 years of age. This clinical study was conducted in accordance with the Declaration of Helsinki. Individuals were limited to patients with a family member who had peg lateralis. Patients with systemic disease or other tooth anomalies were excluded. The participant demographics are presented in Table 1. Additionally, saliva samples were collected from the family members of three individuals with peg lateralis, who voluntarily agreed to participate, to further validate the association between candidate variants and peg lateralis.

**TABLE 1:** Demographics of the study participants.

Pt	Sex	Age	Site	Family history
1	M	23	Both	Mother
2	F	22	Both	Mother
3	M	44	Both	Son
4	F	18	Right	Mother
5	F	61	Left	Sister, Son
6	F	23	Right	Father
7	F	42	Right	Daughter
8	F	33	Right	Sister
9	M	24	Right	Father
10	F	19	Right	Mother
11	F	20	Both	Aunt
12	F	40	Both	Cousins
13	F	24	Both	Aunt, Cousins
14	M	25	Both	Parents
15	M	17	Right	Mother
16	F	44	Both	Son, Mother
17	M	21	Both	Brother
18	F	54	Both	Son
19	M	23	Right	Uncle
20	F	30	Left	Mother, Brother

For DNA analysis, saliva samples were collected from the individuals using a DNA self-collection kit (DNA Genotek, Ottawa, Ontario, Canada). Each individual provided 2 mL saliva in the collection kit containing a 2 mL DNA-preserving solution in the lid. After collecting saliva, the lid on the kit was closed, and the DNA-preserving solution was mixed with the saliva.

Control data were obtained from Asan cohort, which was provided by the Korea Biobank, Center for Genome Science, National Institute of Health, Korea Centers for Disease Control and Prevention. The cohort included genomic and biospecimen data from 5,012 adults aged 40–69 years residing in Ansan, South Korea. Even though the sample size was small, to ensure comparability with the case group, 100 individuals were randomly selected without stratification by sex, age, or major medical history, thereby minimizing selection bias and preserving the natural allele frequency distribution representative of the general population. To further evaluate the representativeness of this control subset, we compared allele frequencies with those of the East Asian population in gnomAD and observed a strong correlation (Pearson’s R = 0.88; Spearman’s ρ = 0.87), supporting the validity of this control group (Supplementary Figure S1).

### 2.2 WES data variant filtering using statistical analysis

Variants were identified from the WES results. Initial filtering excluded variants with low predicted functional impact, including those annotated as LOW or MODIFIER impact by SnpEff or predicted via *in silico* mutation impact analysis (Cingolani et al., 2012). Additionally, only variants more frequently observed in the patient group than in controls were retained. This resulted in 12,771 variants, for which Fisher’s exact test was performed by comparing allele counts between 100 controls and 20 patients. The resulting p-values were adjusted using false discovery rate (FDR) correction with the Benjamini–Hochberg method. Variants with a minor allele frequency (MAF) ≥ 5% in the East Asian population or with no reported MAF were then excluded using the genome aggregation database (gnomAD) v2.1.1 dataset (Karczewski et al., 2020). Among the remaining variants, protein-coding variants with an FDR <10^–10^ are listed in Table 2, and those with an FDR <0.01 are presented in Supplementary Table S1.

**TABLE 2:** List of peg lateralis-related variants after filtration with statistical analysis and *in silico* mutation impact analysis.

Gene	dbSNP_id	UniProt ID	Transcript ID	CHR - POS - REF - ALT	Mutation type	PolyPhen2	SIFT	CES	SnpEff impact	EST ASN MAF	MAF
*11-131H24.4*	No_id	No_id	ENST00000557646	14–94410275 - CAA-CA, C	Frameshift variant p.Lys57fs				High	ND	ND
*OTOP1*	rs199742451	Q7RTM1	ENST00000296358	4–4190576 - C-G	Missense variant p.Arg598Pro	PD	D	I	Moderate	0.01047	0.006169

Statistical analysis used the Ansan cohort as the control. The impacts of the mutation on canonical transcripts were predicted using SIFT, PolyPhen2, CES, and SnpEff.

dbSNP_id, variant ID, in dbSNP; UniProt ID, protein ID, in UniProt; Transcript ID, canonical transcript ID; CHR, chromosome; POS, base pair position; REF, reference allele; ALT, alternative allele; Effect, mutation type, types of mutation in protein; PolyPhen2, mutation impact predicted by PolyPhen2. ‘PD’, probably damaging; SIFT, mutation impact predicted by SIFT. ‘D’, damaging; CES, mutation impact predicted by CES. ‘I’, intolerant; SnpEff Impact, categorized mutation impact pre-defined by sequence ontology effect; EST ASN, MAF, alternative allele frequency in gnomAD, v2.1.1 east asian descendent samples; MAF, alternative allele frequency in whole gnomAD v2.1.1 samples.

### 2.3 *In-silico* mutation impact analysis

An *in silico* mutation impact analysis was performed to identify potentially pathogenic mutations in patients with peg lateralis. Missense variants in all possible non-redundant protein sequences of Ensembl GRCh37.p13 were analyzed using Polymorphism Phenotyping v2 (PolyPhen2), SIFT, and an integrated score of co-evolution and conservation (CES) (Adzhubei et al., 2010; Kim et al., 2019; Vaser et al., 2016). These methods predict the effects of mutations by analyzing evolutionary patterns in protein sequences. We obtained PolyPhen2 and SIFT scores for candidate variants from SnpEf, which uses pre-calculated scores from the DbNspf database (Cingolani et al., 2012; Liu et al., 2020). The CES score was measured using pre-calculated scores provided on the CES website (https://sbi.postech.ac.kr/w/CE). The results of mutation impact analysis are summarized in Table 2.

### 2.4 *In-silico* analysis of mutation impact on protein structure

To investigate the impact of the Arg598Pro (Arginine 598 to proline) mutation on the protein structure of otopetrin-1 (Otop1), we generated a 3D structure of Otop1 using AlphaFold2, a state-of-the-art tool for predicting protein structure (Jumper et al., 2021). We selected interacting residues in which the shortest distance from Arg598 was less than 4 Å. Naccess was used to quantify the relative solvent accessibility (RSA) of the residues. Naccess calculates the atomically accessible surface by simulating the motion of a solvent molecule over a van der Waals surface (Lee and Richards, 1971). Changes in the energy of residues (ΔΔG) upon mutation were assessed using the FoldX and PyRosetta computational methods (Chaudhury et al., 2010; Schymkowitz et al., 2005). For FoldX, we employed the BuildModel function in its mutation engine to predict stability changes, and the default scoring function was used for PyRosetta.

### 2.5 Known hypodontia variants in Korean peg lateralis patients

To investigate the association between peg lateralis in a Korean cohort and known hypodontia-related variants, we performed a targeted variant analysis. Sequencing data from the cohort were examined for variants associated with hypodontia, using the DisGeNet database as a reference for known hypodontia-related genetic variants (Pinero et al., 2020). Statistical analysis was conducted to assess any significant associations between the identified variants and peg lateralis. Our findings revealed no significant associations with known hypodontia-related variants, suggesting that peg lateralis in our cohort is not linked to previously identified hypodontia-related genes.

## 3 Results

### 3.1 Identification of candidate variants discovered via WES analysis

We identified two candidate variants via WES analysis of 20 individuals with peg lateralis (Table 2). One of these variants was a rare variant, rs199742451, in *OTOP1*, with an MAF of 0.0105 in the East Asian population. All 20 individuals with peg lateralis had a heterozygous genotype for this variant; however, none of the 100 control individuals carried this variant. The *P*-value of Fisher’s exact test was 1.18 × 10^−18^, and the FDR-corrected p-value was 2.19 × 10^−15^. The other candidate variant, a frameshift in *RP11-131H24.4*, was not analyzed owing to a lack of available literature.

### 3.2 *In-silico* mutation impact analysis of the *OTOP1* variant

**FIGURE 2:**
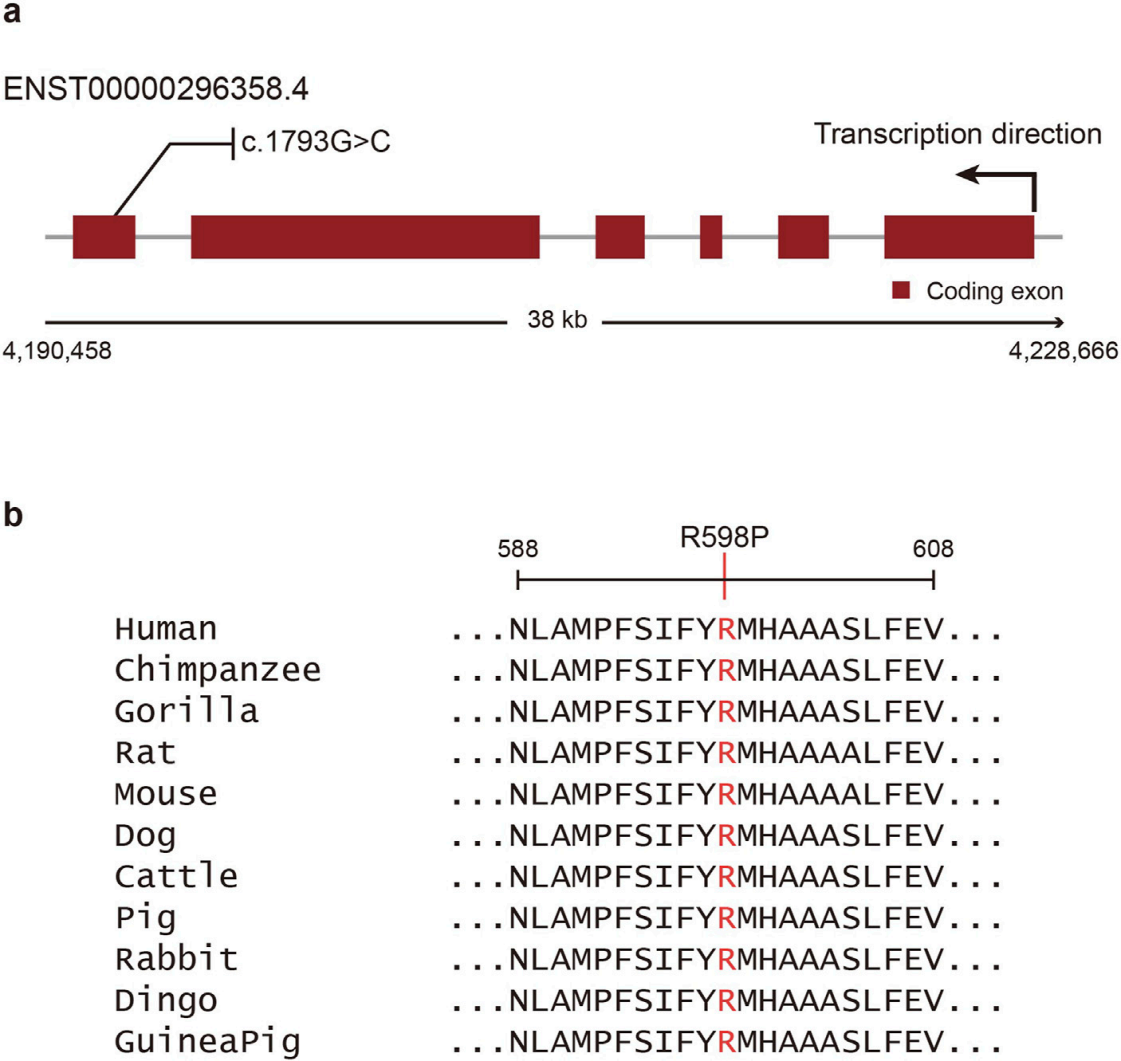
Schematic view and multiple sequence alignments of Otop1. **(a)** Coding exons are shown as red boxes, and introns are shown as gray lines. The missense variant (c.1793G>C, p.Arg598Pro, rs199742451) identified herein is shown above the exons. **(b)** Multiple sequence alignments for Otop1 homologs in various mammal species. The mutated Arg598 residue is indicated using red font.

In-silico mutation impact analysis predicted that the missense variant of *OTOP1* (c.1793G>C, p.Arg598Pro, rs199742451) is deleterious. Three different tools were used for the analysis: Polyphen2 (Adzhubei et al., 2010), SIFT (Vaser et al., 2016), and CES (Kim et al., 2019). All three tools predicted that the *OTOP1* variant is probably damaging and intolerant (Table 2). Additionally, a recently developed tool, AlphaMissense (Cheng et al., 2023), also predicted the variant to be pathogenic with a high confidence score of 0.9765 (Supplementary Figure S1). These predictions suggest that the variant is evolutionarily conserved and may be under strong selective pressure. Evolutionary analyses implied that the observed variant would likely give rise to a dysfunctional Otop1 protein. As shown in Figure 2a, the missense variant is located in exon 1 of *OTOP1*, and the arginine at amino acid position 598 in this exon is well conserved across mammalian orthologs (Figure 2b).

### 3.3 Potential effect of OTOP1 mutation on protein 3D structure

**FIGURE 3:**
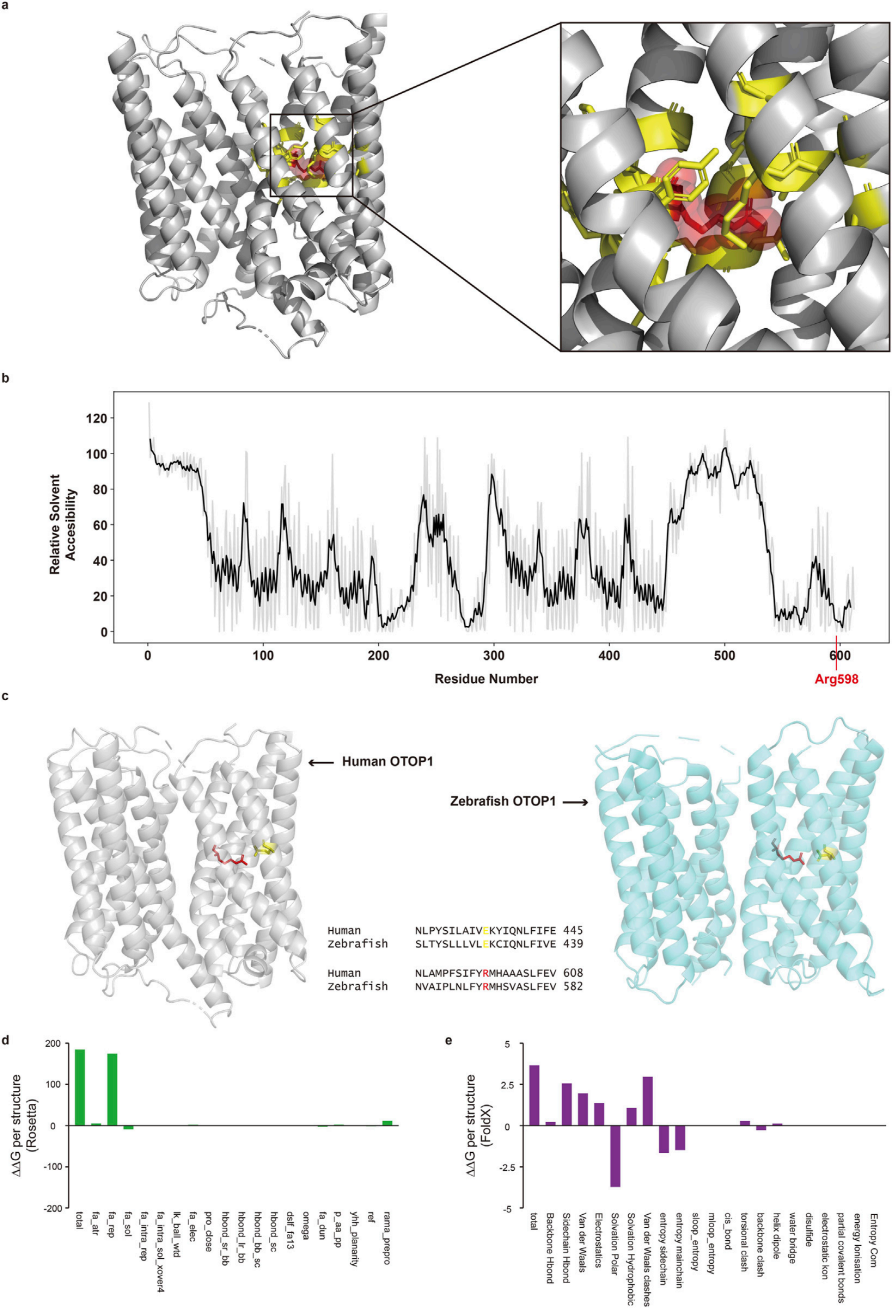
Protein structural analysis of the Otop1 Arg598Pro variant. **(a)** Three-dimensional structure of Otop1 predicted by AlphaFold. The structure was rendered using PyMO (residues with pLDDT<50 are hidden). The red spheres and yellow sticks indicate arginine 598 (Arg598) and its interacting amino acid residues, respectively. The Arg598 residue interacts with 16 residues. **(b)** Relative solvent accessibility (RSA) plot of residues in Otop1. The black line depicts the mean RSA of −2 to +2 residues. **(c)** Comparison of structural characteristics of the Otop1 Arg598Pro variant and sequence alignment between human and zebrafish homologs. The gray structure is the three-dimensional protein structure of human Otop1 predicted by AlphaFold. The blue structure is the three-dimensional protein structure of zebrafish Otop1 (PDB ID: 6NF4). The red and yellow residues in human Otop1 indicate arginine 598 (Arg598) and glutamic acid 435 (Glu435), respectively. The red and yellow residues in zebrafish Otop1 are the corresponding sites of human Otop1Arg598 and Glu435, respectively. The structure was rendered using PyMOL. **(d)** Energy changes calculated with Rosetta and broken down by each score term. **(e)** Energy changes calculated with FoldX and broken down by each score term. fa_atr, Lennard-Jones attractive between atoms in different residues; fa_rep, Lennard-Jones repulsive between atoms in different residues; fa_sol, Lazaridis-Karplus solvation energy; fa_intra_rep, Lennard-Jones repulsive between atoms in the same residue; fa_intra_sol_xover4, Intra-residue Lazaridis-Karplus solvation energy; lk_ball_wtd, Asymmetric solvation energy; fa_elec, Coulombic electrostatic potential with a distance-dependent dielectric; pro_close, Proline ring closure energy and energy of psi angle of preceding residue; hbond_sr_bb, Backbone-backbone hbonds close in primary sequence; hbond_lr_bb, Backbone-backbone hbonds distant in primary sequence; hbond_bb_sc, Sidechain-backbone hydrogen bond energy; hbond_sc, Sidechain-sidechain hydrogen bond energy; dslf_fa13, disulfide geometry potential; omega, omega dihedral in the backbone. A harmonic constraint on planarity with standard deviation of omega dihedral in the backbone; fa_dun, Internal energy of sidechain rotamers as derived from Dunbrack’s statistics; p_aa_pp, Probability of amino acid, given torsion values for phi and psi; yhh_planarity, A special torsional potential to keep the tyrosine hydroxyl in the plane of the aromatic ring; ref, Reference energy for each amino acid. Balances internal energy of amino acid terms. Plays role; rama_prepro, Ramachandran preferences (with separate lookup tables for pre-proline positions and other posit.). (Explanation for the x-axes is from the Rosetta and FoldX official websites).

*In-silico* structural analysis indicated that the Arg598Pro mutation is likely to have a deleterious impact on protein function. Analysis based on the tertiary structure of the protein showed that the Arg598 residue had limited exposure to solvents and interacted with 16 other amino acids (Figure 3a, yellow sticks). The RSA value of Arg598 was low (Figure 3b), suggesting that the residue is located within the inner region of the protein and has limited exposure to solvent. Therefore, the Arg598Pro substitution, where Arg is substituted by an amino acid with very different physicochemical properties at a low RSA position, may interfere with proper protein folding. In addition, the Arg598Pro variant was predicted to reduce protein stability. Both Rosetta and FoldX, which calculate the stability change produced by mutations using the 3D structure of the protein, predicted that the Arg598Pro mutation makes OTOP1 less stable. The total stability changes predicted by Rosetta and FoldX were 184.533 rosetta energy units and 3.6573 kcal/mol, respectively (Figures 3d,e). Furthermore, the reduced protein stability caused by mutations can potentially interfere with protein function.

The Arg598Pro substitution in Otop1 disrupts a direct interaction with Glu435, which may impair protein function. The interaction between the aligned sites with Arg598 and Glu435 was critical for the proton transport activity of zebrafish Otop1 (Saotome et al., 2019). A single mutation that interferes with the electrostatic interaction between Arg598 and Glu435 greatly diminished the current amplitude observed from Otop1. The tertiary structures of human Otop1 and zebrafish Otop1 are well conserved, with the conformation and interactions of Arg598 and Glu435 maintained in both species (Figure 3c). This suggests that changes in the interaction between Arg598 and Glu435 may also lead to the loss of protein function in human OTOP1.

### 3.4 Functional analysis of *OTOP1*

Functional analysis of *OTOP1* suggested a possible association with peg lateralis. Gene ontology terms associated with *OTOP1*, obtained from the AmiGO database, included biomineral tissue development, which is critical for tooth development (Gene Ontology, 2021; Moradian-Oldak and George, 2021). This suggests a possible relationship between *OTOP1* and the development of peg lateralis.

### 3.5 Validation of the *OTOP1* variant in additional patients

**FIGURE 4:**
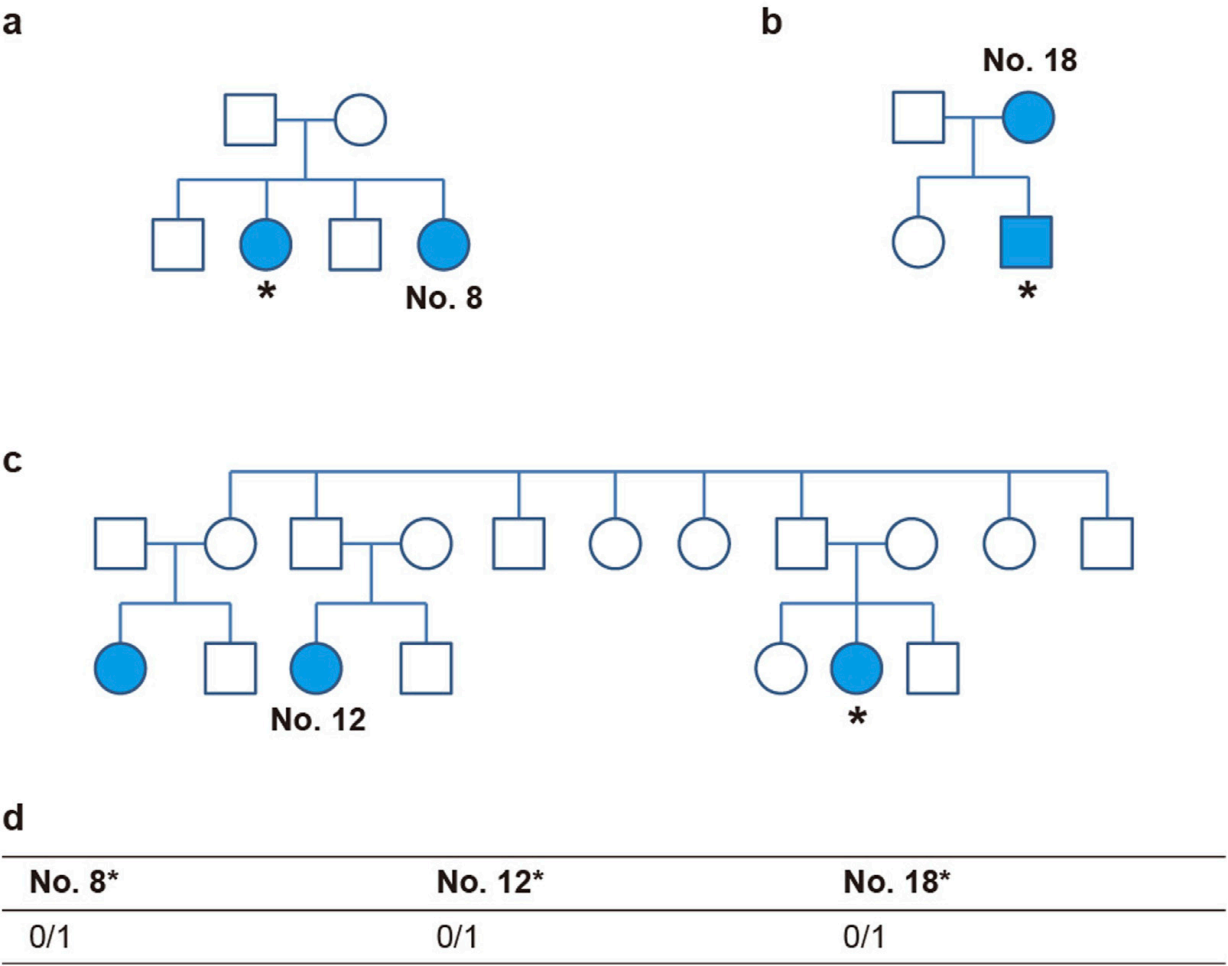
OTOP1 genotype of family members of three individuals. **(a–c)** Pedigrees of family No. 8 **(a)**, No. 18 **(b)**, and No. 12 **(c)**. Individuals with diseases are indicated by colored markers. The asterisk (*) denotes a subject who underwent additional sequencing. **(d)** The table shows the rs199742451 genotypes of three individuals.

To further validate the relationship between the candidate gene and peg lateralis, the genotype of loci was additionally identified in the family members of study participants, as shown in Figure 4. Genetic analysis was performed only in relatives who exhibited the peg lateralis phenotype. Sequencing the samples from family members of three individuals (the sister of No. 8, cousin of No. 12, and mother of No. 18) revealed the same heterozygous variants as found in the 20 study participants. This finding suggests that the variants are associated with the occurrence of peg lateralis within the family (Figure 4).

### 3.6 Known hypodontia variants in Korean peg lateralis patients

The presence of peg lateralis is closely linked to other dental irregularities, including hypodontia, the genetic basis of which is relatively well-studied (Baccetti, 1998). None of the study participants exhibited any association with variants known to cause hypodontia. Analysis of hypodontia-related variants obtained from the DisGeNet database did not show any significant association (Table 3) (Pinero et al., 2020). This suggests that the peg lateralis in our cohort is not related to previously identified hypodontia-related genes.

**TABLE 3:** Statistical significance of known hypodontia-related variants in a Korean peg lateralis cohort.

Gene	dbSNP_id	CHR	POS	REF	ALT	Fisher_p	Allele count				Reference
							con_ref	con_alt	pat_ref	pat_alt	
*WNT10A*	rs147680216	2	219754966	G	A	0.194875	197	3	38	2	Parveen et al. (2019)
*GLI3*	rs929387	7	42005678	G	A	0.813271	31	167	7	33	Liu et al. (2013)
*PAX9*	rs12881240	14	37135752	C	T	1	151	43	31	9	Zhang et al. (2014)
*PAX9*	rs4904210	14	37135753	G	C	0.608038	102	94	19	21	Wang et al. (2011)
*PLD2*	rs3764897	17	4722876	G	A	0.21562	180	20	39	1	Mostowska et al. (2011)
*AXIN2*	rs2240308	17	63554591	G	A	0.709915	138	62	26	14	Mártha et al. (2019)

Known hypodontia-related variants were collected from DisGeNET., dbSNP_id, variant ID, in dbSNP; CHR, chromosome; POS, base pair position; REF, reference allele; ALT, alternative allele; Fisher_p, p-value obtained in the Fisher Exact test; con_ref, reference allele count among 100 controls; con_alt, alternative allele count among 100 controls; pat_ref, reference allele count among 20 patients; pat_alt, alternative allele count among 20 patients.

## 4 Discussion

In this study, all patients with peg lateralis carried heterozygous mutations in two candidate loci: *RP11-131H24.4*, an uncharacterized genomic region, and a missense mutation in exon 1 of *OTOP1* (c.1793G>C, p.Arg598Pro, rs199742451) (Figure 3). Otopertrin-1 belongs to the otopetrin family of multi-transmembrane domain proteins that are highly conserved in mammals (Hurle et al., 2003). Otop1 is required for the formation of otoconia and otoliths, with *OTOP1* mutations in mice and zebrafish leading to non-syndromic otoconia agenesis. Otopetrin proteins are proton-selective ion channels activated by purinergic stimuli in vestibule-supporting cells, with crucial roles in regulating intracellular calcium concentration (Hughes et al., 2007). As otoconia are essential for mechanosensory transduction of linear acceleration and gravity in the inner ear, their degeneration or displacement can result in dizziness and progressive loss of balance. Otop1 expression is continuously observed in adult mice and is involved in the maintenance and restoration of otoconia mineralization. However, whether Otop1 expression persists in humans remains unclear (Kim et al., 2010). Although the biochemical role of Otop1 was recently identified, it has not been implicated in odontogenesis. Nevertheless, our results suggest that *OTOP1* and the unknown gene *RP11-131H24.4* are likely to affect the development of a peg-shaped tooth directly or indirectly.

During dental development, ion channels regulate various physiological and biological activities, such as pH control, calcium flux, and gene expression (Duan, 2014). The loss-of-function mutations of Otop1 identified in the present study might cause an influx of calcium ions and disrupt calcium homeostasis, potentially leading to peg lateralis (Figure 4). One study revealed that mutations in *CACNA1S*, which encodes a voltage-dependent calcium channel, leads to the formation of multiple cusps in molars, thus demonstrating that defects in calcium signaling could give rise to variation in tooth morphology (Laugel-Haushalter et al., 2018).

As otopertrin-1 is a proton-selective ion channel, it is activated by acidic conditions and directly gated by protons (Teng et al., 2022). Tain et al. suggested that otopertrin-1 is activated by alkaline conditions, with mutations in Otop1 affecting its activation under these conditions while exerting no effect in acidic conditions (Tian et al., 2023). The alkali-activated Otop1 is permeable to protons and cations but not to calcium ions. Although the mechanism of pH regulation in odontogenesis remains unknown, mutant Otop1 might contribute to abnormal tooth morphology by altering calcium ion concentrations.

Tooth development progresses through the initiation, bud, and bell stages and under the control of several signaling pathways. An important part of tooth development is the folding of the tooth epithelium at the cap and bell stage, regulated by primary and secondary enamel knots (Jernvall and Thesleff, 2012). During tooth morphogenesis, the enamel knot acts as a signaling center, expressing key factors such as Sonic Hedgehog (SHH), Bone Morphogenetic Proteins (BMPs), Fibroblast Growth Factors (FGFs) and Wingless-related Integration Site (WNT) proteins. These signals regulate tooth size and shape by controlling proliferation and folding of the dental epithelium (Jernvall et al., 1994). In addition, apoptosis in the enamel knot determines tooth size or morphology by regulating the duration of signaling interactions and is crucial in determining the size and patterning of cusps (Jernvall et al., 1998). Kim et al. demonstrated that inhibiting the apoptosis of enamel knots reduced the crown height (Kim et al., 2006). The enamel knot affects the number, shape, size, and location of cusps, thus determining the shape of the occlusal surface of molars. Consequently, the final shape of the crown is already determined at the bell and cap stages (Jernvall and Thesleff, 2012).

*CACNA1S* is expressed in epithelial cells around the secondary enamel from the initial bud to the postnatal stage. Altering the inflow of calcium near the enamel knots suggests that the molars caused additional cusps. *CACNA1S* is also expressed in Hertwig’s epithelial root sheath during postnatal development and is implicated in mal-shaped roots (Kantaputra et al., 2023). Thus, the morphology of teeth is affected by spatiotemporal gene expression patterns. Furthermore, although the molar exhibited more complex cusps, the premolar had a lower complexity because of mutation. This suggested that this difference could be attributed to variations in tissue sensitivity to mutations (Kantaputra et al., 2023). Therefore, whether the same incisors express the mutant phenotype may depend on these differences. However, animal studies are required to investigate the timing and location of candidate gene expression to confirm their effects on peg-shaped tooth generation.

Herein, we employed WES to analyze the DNA of patients with peg lateralis unassociated with other anomalies or syndromes. We identified *OTOP1* and *RP11-131H24.4* as candidate genes contributing to the development of peg lateralis. Additional analysis within families identified that close family members carried the same genetic variant, which supported the finding from the initial study cohort (Figure 4). In one family, mutation was absent in parents but present in the offspring while in other families, it was observed only in cousins. These observations indicate variable expressivity and incomplete penetrance of the implicated genetic variants. This reflects phenotypic heterogeneity among carriers of identical genotypes and emphasizing the multifactorial and complex genetic architecture underlying dental anomalies (Kim et al., 2017). Additionally, genes previously reported as associated with hypodontia and microdontia were not implicated in the cases analyzed herein (Table 3). Notably, our results contrast with previous findings reporting that dental anomalies within the same genotype exhibit distinct phenotypes. This difference is likely because our study exclusively selected individuals with isolated peg lateralis, even though peg lateralis is more commonly associated with other abnormalities (Kim et al., 2017). Although the roles of *OTOP1* and the other genes identified herein in dental development have not been clearly defined, our results suggest the possibility of an interrelation between these two candidate genes and peg-shaped tooth development. Therefore, the potential function of *OTOP1* in tooth morphogenesis warrants further research.

Although both variants were recurrently identified in 20 Korean patients, the limited sample size precludes definitive conclusions regarding their association with peg lateralis. Analyzing the entire family in future, including members without peg lateralis, could provide stronger evidence for the relationship between the gene and phenotype. Further familial analyses, particularly involving unaffected relatives, will be crucial to substantiate these preliminary findings. Nevertheless, the current study proposes a potential association between these candidate genes and peg lateralis, offering preliminary insight and laying a groundwork for further research into the molecular etiology and developmental mechanisms underlying peg lateralis.

## 5 Conclusion

Herein, we analyzed genotypes associated with non-syndromic and isolated peg lateralis in Koreans using WES. Heterozygous alleles for two candidate genes were identified in all patients. These included an unknown gene *RP11-131H24.4* and *OTOP1,* which encodes a proton-selective ion channel. However, WES and *in silico* analysis alone could not conclusively determine a strong relationship between candidate genes and peg lateralis. This work is a preliminary exploration of genotypes associated with peg-shaped teeth and provides a basis for further research into the matter. Future investigations involving larger cohorts and functional studies carrying Otop1 variants, would be needed to determine the genetic basis of peg lateralis conclusively.

## Data Availability

The raw sequence reads of the cohort can be found in the BioProject PRJNA1013609.
