# Supplementary material for: Genetic analysis of non-syndromic peg lateralis using whole-exome sequencing
Source: Front Genet. 2025 Aug 13;16:1572966. doi: 10.3389/fgene.2025.1572966 (PMC12380783; doi:10.3389/fgene.2025.1572966)
Supplement: Supplementary file 1 [file Supplementaryfile1.docx]

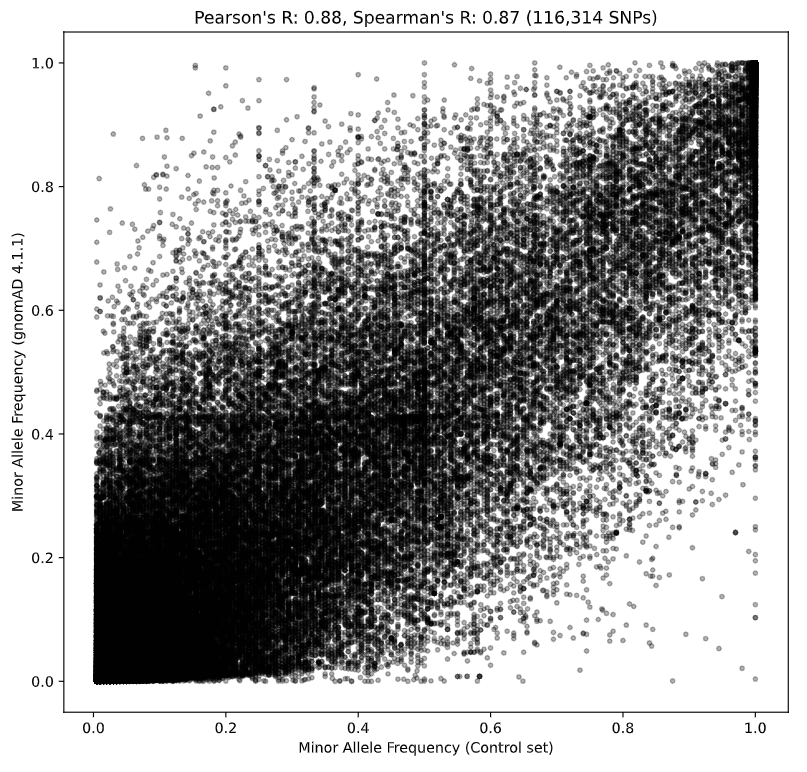


**Supplementary Figure 1.** **Correlation of minor allele frequencies between the control subset and gnomAD East Asian population.**

Scatter plot comparing the minor allele frequencies (MAFs) of 116,314 SNPs observed in our control subset (x-axis) and the East Asian population from gnomAD v4.1.1 (y-axis). Each point represents a single SNP. The strong correlation (Pearson’s R = 0.88; Spearman’s R = 0.87) indicates that the allele frequency distribution in the control subset reasonably reflects that of the broader East Asian population, supporting its use as a reference for allele frequency comparisons in this study.
